# Longitudinal serum proteomics analyses identify unique and overlapping host response pathways in Lyme disease and West Nile virus infection

**DOI:** 10.3389/fimmu.2022.1012824

**Published:** 2022-12-09

**Authors:** Patrick Boada, Benoit Fatou, Alexia A. Belperron, Tara K. Sigdel, Kinga K. Smolen, Zainab Wurie, Ofer Levy, Shannon E. Ronca, Kristy O. Murray, Juliane M. Liberto, Priyanka Rashmi, Maggie Kerwin, Ruth R. Montgomery, Linda K. Bockenstedt, Hanno Steen, Minnie M. Sarwal

**Affiliations:** ^1^ Division of Transplant Surgery, Department of Surgery, University of California, San Francisco, CA, United States; ^2^ Department of Pathology, Boston Children’s Hospital - Harvard Medical School, Boston, MA, United States; ^3^ Precision Vaccines Program, Boston Children’s Hospital, Boston, MA, United States; ^4^ Department of Internal Medicine, Yale School of Medicine, New Haven, CT, United States; ^5^ Division of Infectious Diseases, Boston Children’s Hospital – Harvard Medical School, Boston, MA, United States; ^6^ Broad Institute of Massachusetts Institute of Technology & Harvard, Cambridge, MA, United States; ^7^ Division of Tropical Medicine, Department of Pediatrics, Baylor College of Medicine, Houston, TX, United States; ^8^ William T. Shearer Center for Human Immunobiology, Texas Children’s Hospital, Houston, TX, United States

**Keywords:** West Nile virus, serum proteomics, acute-phase response, immune system, localized and disseminated stage, asymptomatic infection, Lyme disease, longitudinal analysis

## Abstract

Advancement in proteomics methods for interrogating biological samples has helped identify disease biomarkers for early diagnostics and unravel underlying molecular mechanisms of disease. Herein, we examined the serum proteomes of 23 study participants presenting with one of two common arthropod-borne infections: Lyme disease (LD), an extracellular bacterial infection or West Nile virus infection (WNV), an intracellular viral infection. The LC/MS based serum proteomes of samples collected at the time of diagnosis and during convalescence were assessed using a depletion-based high-throughput shotgun proteomics (dHSP) pipeline as well as a non-depleting blotting-based low-throughput platform (MStern). The LC/MS integrated analyses identified host proteome responses in the acute and recovery phases shared by LD and WNV infections, as well as differentially abundant proteins that were unique to each infection. Notably, we also detected proteins that distinguished localized from disseminated LD and asymptomatic from symptomatic WNV infection. The proteins detected in both diseases with the dHSP pipeline identified unique and overlapping proteins detected with the non-depleting MStern platform, supporting the utility of both detection methods. Machine learning confirmed the use of the serum proteome to distinguish the infection from healthy control sera but could not develop discriminatory models between LD and WNV at current sample numbers. Our study is the first to compare the serum proteomes in two arthropod-borne infections and highlights the similarities in host responses even though the pathogens and the vectors themselves are different.

## Introduction

Blood is an easily accessible source to assess health and monitor for disease including infection. Characterizing the serum proteome can provide an assessment of immunological and molecular changes in response to varied antigenic exposures. Unbiased, discovery-based serum proteomics enables detection and quantification of several hundred proteins without the need for *a priori* knowledge of biology. We have utilized mass spectrometry (MS)-based proteomic techniques to improve our understanding of host responses to complex disease states in infection and in allotransplantation (1–5). In this study, we applied MS-based strategies to profile the human serum proteome longitudinally in response to two arthropod-transmitted pathogens: the Ixodes tick transmitted spirochete *Borreliella burgdorferi* that causes Lyme disease (LD) (6) and the mosquito-transmitted flavivirus West Nile virus (WNV). Both pathogens enter the human host when the arthropod vector acquires its bloodmeal through the skin bite site. The clinical presentations of these infections represent a spectrum of disease severity ranging from asymptomatic/mild isolated organ system involvement to severe systemic disease (7–9). The immune responses that underlie these different expressions of disease are incompletely understood. Here we define longitudinal changes in the serum proteomes and associated biological pathways in patients with LD and WNV infections and compare these results to blood sampling from healthy controls, using a depletion-based high-throughput shotgun proteomics (dHSP) pipeline. In parallel, a sample-sparing microtiter plate-based proteomics method, named MStern blotting, was also used to examine changes in selected serum proteins in LD and WNV infections over time (5, 10, 11), with a view to understanding the clinical utility of using this platform for rapid, low-cost, low-throughput sample testing. Each platform identified unique and overlapping proteins to better understand host response pathways in the acute and recovery phases of infection with each pathogen. To our knowledge, a comparison of the serum proteomes of these two human infections, occurring from different pathogen types, from a bacterium for LD and from a virus for WNV, has not been reported.

## Method

### Cohort design and patient characteristics

#### Ethics statement

The Lyme disease (LD) and West Nile virus (WNV) cohorts were obtained with written informed consent under the guidelines of the Human Investigations Committee of Yale University School of Medicine (LD) and Baylor College of Medicine (WNV).

**Figure 1:**
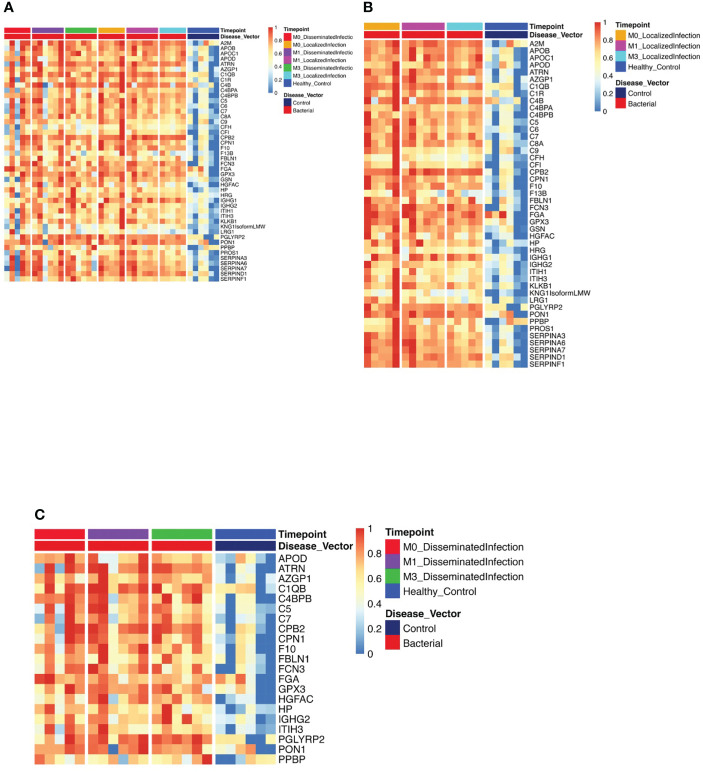
Protein Expression across Lyme Disease Timepoints. Serum samples from LD patients were assessed by the dHSP platform. The limma package was used to identify differentially expressed proteins in a combined dataset of healthy controls and patients with statistically significant proteins defined with FDR ¾= 0.05. **(A)** Normalized heatmap displays the progression of abundant proteins (n=46) in both disseminated and localized infections at month 0, 1, and 3 (M0, M1, M3) compared to healthy controls. **(B)** Normalized heatmap displays the progression of abundant proteins (n = 66) in localized infections at month 0, 1, and 3 (M0, M1, M3). **(C)** Normalized heatmap displays the progression of abundant proteins (n = 22) in disseminated infections at month 0, 1, and 3 (M0, M1, M3).

**Figure 2:**
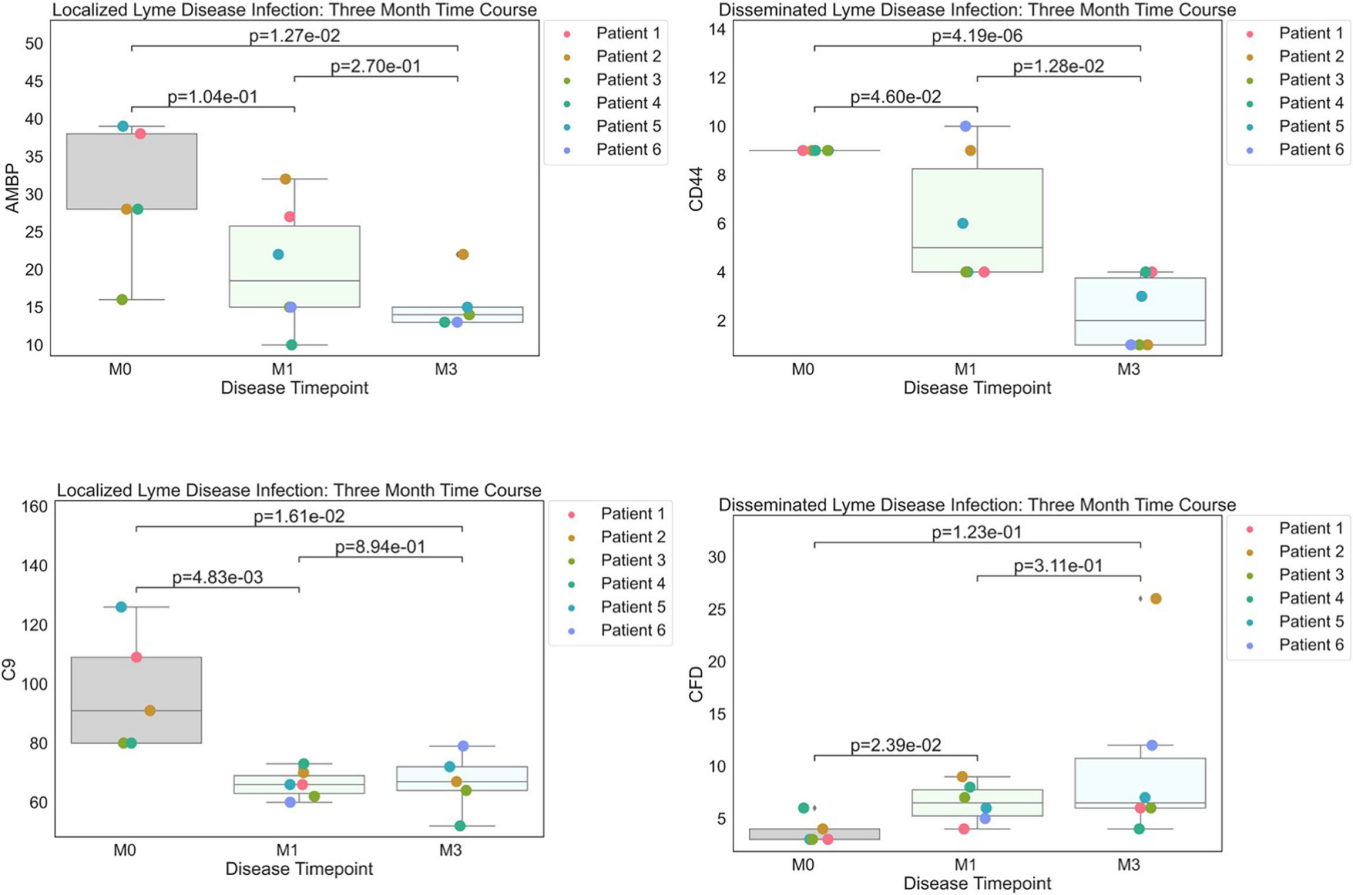
Strip plots of differentially expressed proteins between primary acute infection and recovery in Lyme disease. Serum samples from LD patients were assessed by the dHSP platform. Differentially expressed proteins were identified in a combined dataset of acute infection and convalescent time points. Patients with statistically significant proteins were defined with p value ¾= 0.05. Localized Lyme disease infections were statistically significant across time points for AMBP and C9. CD44 was statistically significant across time points in disseminated Lyme Disease infections, however, CFD was statistically significant by month 1.

LD patients presenting with acute illness to their physicians in CT were recruited and enrolled by study staff at Yale School of Medicine (New Haven, CT) or at Mansfield Family Practice in Storrs, CT. The 12 participants diagnosed with LD were stratified into 2 groups: those with a single erythema migrans (EM) lesion (localized EM; n = 6) and those with clinical signs of dissemination infection (disseminated LD; n = 6). The disseminated LD group included neurologic disease, systemic flu-like illness that was confirmed by seroconversion, carditis and arthritis. Serum samples were collected at 3 different time points: 1) early after diagnosis, range 0-9 days (defined as M0), 2) at convalescence, 30 days post diagnosis (defined as M1), and 3) up to 4.5 months post diagnosis, range 3-4.5 months (defined as M3). Additional details can be found in **Table 1**. For M0 participants enrolled in the study within 9 days after diagnosis; 7 of the 9 participants had received at least one dose of antibiotics (doxycycline) prior to the blood draw. The second time point blood samples (M1; n = 12) were obtained about one month after the initial diagnosis and in all cases after completion of the initial course of antibiotic therapy. The last time point (M3, also defined as “Resolved” in **Figure 1**, **2**; n = 12) was collected 3 to 4.5 months after completion of antibiotic therapy and corresponds to a return to pre-infection clinical baseline as determined by resolved presenting signs and symptoms of the disease.

**Table 1:** Patient demographics (LD and WNV).

Clinical cohort demographics					
	Lyme (Localized EM)	Lyme (Disseminated)	WNV (Asymp)	WNV (Symp)	Healthy Controls
Number of Subjects	6	6	4	7	6
Male	3 (50%)	3 (50%)	4 (100%)	5 (71%)	3 (50%)
Female	3 (50%)	3 (50%)	0 (0%)	2 (29%)	3 (50%)
Mean Age (yr) (SD)	58.2 ± 17	57.5 ± 22.5	67.8 ± 7.4	52.9 ± 14.0	49.5 ± 24.3

At the M0 time point clinical data was available for 5/6 localized EM and 4/6 disseminated LD subjects.

Statistical analysis for age was performed using a two-tailed t-test; and for gender using Chi-square test.

For WNV, patients infected with WNV were recruited at Baylor College of Medicine within seven days of symptomatic onset with infection confirmed by RT-PCR (12). Viremic asymptomatic donors were identified by screening at Gulf Coast Regional Blood Center donation sites (Houston, TX). Symptomatic and asymptomatic subjects were enrolled within ~1 week of infection as confirmed by PCR detection of viremia which becomes undetectable by ~14 days (13). Asymptomatic donors had no acute illness and took no antibiotics or nonsteroidal anti-inflammatory drugs at the time of sampling (14). Medical history was obtained and blood collected at the time of enrollment and at 3 month and one-year follow-up visits.

### MStern 96-well sample processing/digestion and cleanup

Sample processing employed an MStern blotting protocol we have developed as described previously (5, 11). The name MStern blotting is derived from Western blotting as it uses the same type of hydrophobic polyvinylidene fluoride (PVDF) membrane for protein retention. In brief, 1 µL of serum (~50 µg of proteins) was mixed in 100 µL of urea buffer. Following reduction and alkylation of the cysteine side chains, 10-15 µg of proteins were loaded on to a 96-well plate with a PVDF membrane at the bottom (Millipore-Sigma), which had been previously activated and primed. Trypsinization of the proteins adsorbed to the membrane was achieved by incubation with the protease for 2h at 37°C. Resulting tryptic peptides were eluted off the membrane with 40% acetonitrile (ACN)/0.1% formic acid (FA). The peptides were subsequently cleaned-up using a 96-well MACROSPIN C18 plate (TARGA, The NestGroup Inc.).

### dHSP sample preparation

Serum samples were depleted for abundant proteins using HighSelect Top14 Depletion Midi Spin Columns (Thermo Scientific, Cat #A36371) followed by acetone precipitation. Precipitated protein was resuspended in 6M urea-tris buffer. Reduction with DTT followed by alkylation with iodoacetamide was done at room temperature for 1hr. Resulting protein samples were digested with trypsin (1:50 trypsin-to-substrate ratio) overnight at 37 °C and reaction was quenched with trifluoroacetic acid at a final concentration of 0.1%.

### DIA sample acquisition

For MStern platform, the samples were analyzed on the same LC/MS system as the data-dependent acquisition (DDA) runs using identical LC parameters (45 min gradient, 59 min total runtime). The m/z range 375−1200, covering 95% of the identified peptide, was divided into 15 variable windows based on density, and the following parameters were used for the subsequent DIA analysis: resolution 35000 @ m/z 200, AGC target 3e6, maximum IT 120 ms, fixed first mass m/z 200, NCE 27. The DIA scans preceded an MS1 Full scan with identical parameters yielding a total cycle time of 2.4s.

### dHSP data acquisition

Provided samples were acidified with formic acid (final concentration of 2%) to bring the pH < 4 and clean up with C18 Monospin columns. Dried samples were reconstituted in 20 µl reconstitution buffer (2% acetonitrile with 0.1% Formic acid) and 2µl (1µg) of it was injected on the instrument. Mass spectrometry experiment was performed using Q Exactive HF-X Hybrid Quadrupole - Orbitrap mass spectrometer (Thermo Scientific, San Jose, CA) with liquid chromatography using a Nanoacquity UPLC (Waters Corporation, Milford, MA). For a typical LCMS experiment, a flow rate of 300 nL/min is used, where mobile phase A was 0.2% formic acid in water and mobile phase B was 0.2% formic acid in acetonitrile. Peptides were directly injected onto 50cm µPAC (PharmaFluidics) analytical column with a fused silica tip emitter using a gradient (2-38% B, followed by a high-B wash) of 120min. The mass spectrometer was operated in a data dependent fashion using HCD fragmentation for MS/MS spectra generation.

The RAW data were analyzed using Byonic v3.7.13 (Protein Metrics, Cupertino, CA) to identify peptides and infer proteins. A concatenated FASTA file containing the Uniprot *Homo sapiens* sequences from which likely contaminants and impurities were removed before analysis. Proteolysis with Trypsin was assumed to be semi-specific allowing for N-ragged cleavage with up to two missed cleavage sites. The precursor ion tolerance was set to 12 ppm. The fragment ion tolerance was set to 0.4 Da. Cysteine modified with propionamide was set as a fixed modification in the search. Variable modifications included oxidation on methionine, histidine and tryptophan, dioxidation on methionine and tryptophan, deamidation on asparagine, and acetylation on protein N-terminus. Proteins were held to a false discovery rate of 1% using standard reverse-decoy technique (15).

### Spectral library generation and DIA data analysis

We use a previously published in house generated spectral library (5). All DIA data were directly analyzed in Spectronaut v12.0.20491.18 (Biognosys, Switzerland). Standard search settings were employed, which included enabling dynamic peak detection, automatic precision nonlinear iRT calibration, interference correction, and cross run normalization (total peak area). All results were filtered by a q-value of 0.01 (corresponding to an FDR of 1% on the precursor and protein levels). Otherwise default settings were used.

### MStern statistics and data analysis

The protein abundance/samples matrix was exported into Perseus (16) where a log2 transformation followed by imputing the missing values for each column from a normal distribution (width = 0.3; down shift = 1.8). For better visualization, the results were exported into GraphPad Prism software v8. The area under the ROC curve (AUROC) calculation for the biomarker panels was done using IBM’s SPSS software. To minimize between subject variation, we used the last time point (M3) as “Resolved” as patients at this time point have resolved their acute infection (17, 18). Given the pilot nature of this study and limited number of samples, no multiple testing corrections were applied. The DEPs were exported into STRING protein-protein interaction network to assess their functional relationships (19). We used the short time-series expression miner (STEM) tool to visualize protein profiles and dynamics among the 3 time points of localized LD and disseminated LD separately (20). The averaged protein intensities were calculated for each time point and then the data were normalized such that the time point M0 was set to 0. Data completeness (**Figure S1D**). The resulting complete matrix was comprised of 295 protein entries. Eighty-seven percent (n = 259) of the detected protein groups were present in at least half of the samples, and 63% (n = 185) of the detected protein groups were found in all samples. Overall, only 13% of the protein quantitation values across all samples were missing, which is consistent with published DIA-based proteomics studies (21–24).

For verification of LD results with Zhou et al. (7), we compared M0 and M3 samples, defined as Lyme and “Resolved”, respectively (**Figure 2**). The M3 time point was considered the most appropriate comparison in our cohort because it is closest to the individual’s immune baseline and these patients reported resolution of LD signs and symptoms (17, 18).

### dHSP preprocessing

Data preprocessing was conducted with Python (3.8.8), Pandas (1.2.0), Numpy (1.19.5), and Re (2.2.1). The dHSP pipeline identified a total of 2086 (LD) and 2091 (WNV) proteins in sera with a platform identification capability of 4389 total proteins. The data was set at an 20% limit for missing data. Any aberrated or missing values were imputed by the minimum value observed in a similar cohort member. Furthermore, all raw abundance data was log(2) normalized prior to any further analysis. All exploratory data analysis and descriptive statistical plots were generated by using Seaborn (0.11.1). Original patient labeling was obscured and reclassified programmatically by using the regex package in Python.

### dHSP differential expression analysis

Differential Expression Analysis was conducted by linear models by using R (4.0.4), limma (3.46.0) and ggplot2 (3.3.5). Comparisons were created to discern a WNV or LD signature by comparing differences between initial acute disease timepoint and healthy control readouts. Statistical significance was accepted with a FDR less than or equal to 0.05. Corresponding results were illustrated as heatmaps using pheatmap (1.0.12)

### dHSP descriptive statistics and temporal analysis

Proteins with the greatest log fold change between initial acute infection and recovery through linear models (limma 3.46.0) were observed for temporal perturbations. Moreover, descriptive statistics were captured by using the Python packages matplotlib (3.3.4), numpy (1.19.5), pandas (1.2.0), scipy (1.7.2), and seaborn (0.11.1). Comparisons for disease signature were defined by observing difference between the initial acute disease timepoint and the final recovery timepoint. Furthermore, t-tests were run for pairwise comparisons between M0 & M1, M1 & M_final_, and M0 & M_final_.

### dHSP random forest

To further analyze distinct differences between patient Viral and Bacterial infection status data science and machine learning methods were utilized. A random forest machine learning model (Python 3.8.8, Sklearn: 0.24.1) was utilized to classify patients who are WNV+ or LD+. Moreover, WNV+ and LD+ timepoints were then label encoded for classification to compare against a healthy control cohort. The data was then normalized by standard scaling and mean and standard deviation were computed by fit transformation (Sklearn: 0.24.1). Data was then split to preserve 30% data for testing. A param grid of learning_rate, min_child_weight, gamma, subsample, n_estimators, colsample_bytree, and max_depth was automated by using a randomized search cv. The random search was composed of 50 iterations, scoring based on roc_auc, 4 jobs and a cross validation composed of stratified k fold search of 10 splits. Prediction Accuracy, CV score and AUC were used as metrics to observe the accuracy and validity of the model. Moreover, top contributing features were extracted from machine learning model and evaluated by feature importance score.

### Pathway analysis of shared proteomic readouts between platforms

The set of unique and common proteins between each mass spectrometry platform was run through STRING DB (functional protein association networks) to determine the GO Biological Processes, Molecular Functions, and proteomic interactions.

## Results

### Study design

**Figure 3:**
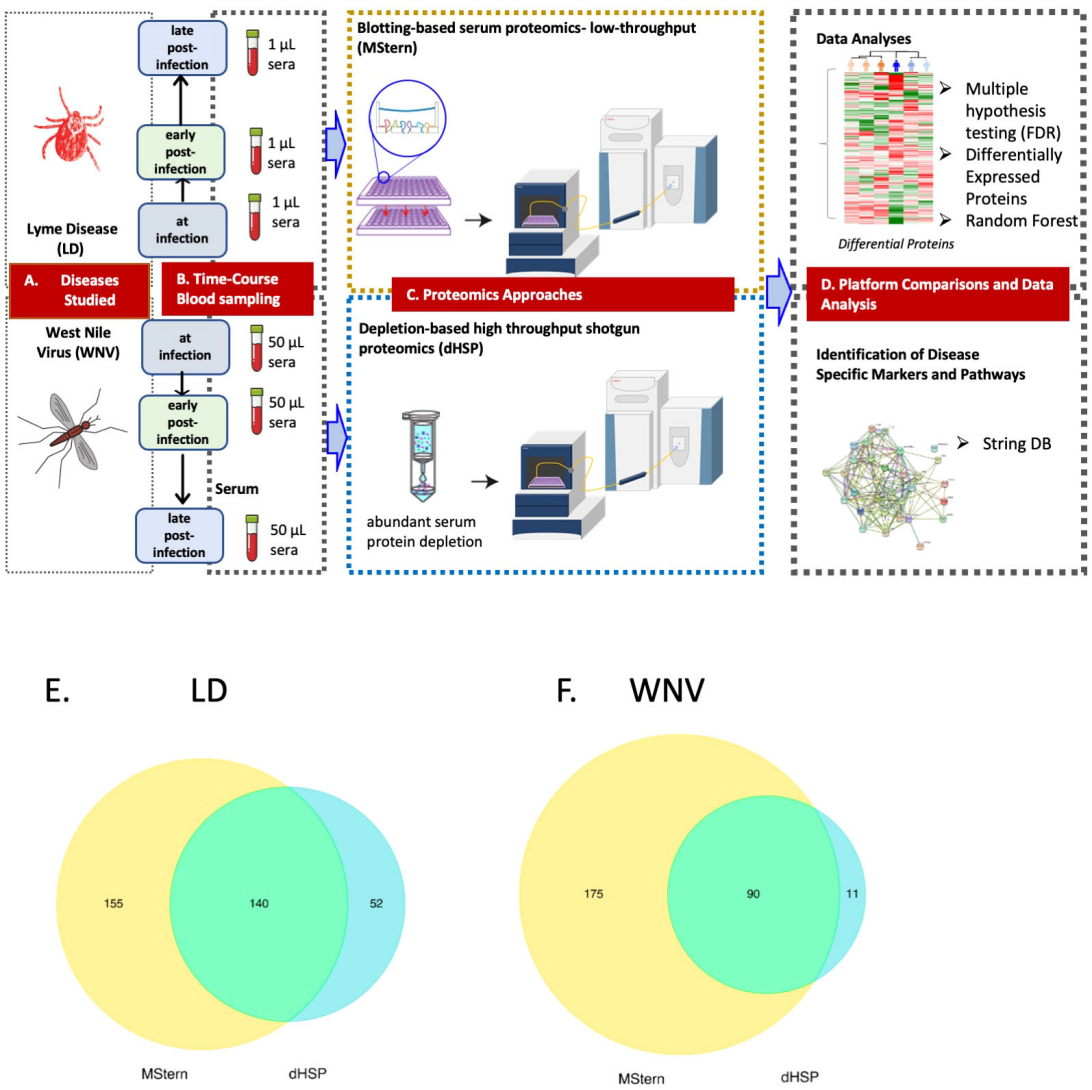
LEGEND - Schematic representation of the overall study. **(A)** Diseases studied- Lyme disease (tick-borne) and West Nile Virus infection (mosquito-borne).**(B)** Blood collection by venipuncture and serum separation **(C)** Serum protein processing platforms– MStern spotting of one µl serum on PVDF microtiter membrane without depleting high abundant protein followed by protein denaturation, reduction and alkylation of cysteine residues, rapid protein digestion, LCMS (top panel) and dHSP with depletion of high abundance blood proteins using spin columns followed by protein denaturation, reduction and alkylation of cysteine residues, rapid protein digestion and LCMS shotgun proteomics (bottom panel) **(D)** Data analysis and data interrogation for marker discovery and underlying molecular mechanisms. Venn Diagram of the analyzed proteins from both the dHSP and MStern platforms in LD **(E)** and WNV **(F)**. The optimized depletion-based method is an unbiased approach that was based on shotgun proteomics method that required 50 μl serum for depletion step and 1μg of tryptic peptides for LC/MS analysis. The MStern platform starts with 1μl serum on a 96 well-plate-based method with no depletion step required in WNV.

Serum samples, extracted from peripheral blood samples obtained by venipuncture, from subjects with LD (n=12) or WNV infection (n=11) were collected at initial diagnosis and at two timepoints after diagnosis: for LD at 1- and 4-6 months convalescence and for WNV at 3- and 12- months convalescence (**Figure 3**). Characteristics of the subjects are described in **Table 1**. LD subjects included patients with localized (n=6) or disseminated disease (n=6); WNV subjects included symptomatic inpatients with clinically worse symptomatology (n=7) and asymptomatic viremic, outpatient subjects (n=4). Samples from healthy controls (HC, n=6, 3 male, 3 female) were assessed at a single time point. Aliquots of samples were processed using established protocols using the depletion-based high-throughput shotgun proteomics (dHSP) pipeline (4, 25) and the non-depleting MStern blotting-based proteomics platform (10) (**Figure 3**).

### Longitudinal proteomic profiles of responses in LD

The dHSP pipeline identified a total of 2086 proteins in sera of subjects with LD with a platform identification capability of 4389 total proteins (26). After data processing, removing proteins with 20% missing data across all samples and including proteins with known biological function, 192 unique proteins were utilized for further analysis. The MStern platform identified a total of 295 proteins on the platform, of which 140 were also detected with dHSP (**Figure 3B**). Gene ontology and molecular function pathways common between the two platforms are shown in **Tables 2A, B**.

**Table 2:** Proteomic Pathways in LD.

A	
Description	False Discovery Rate
Regulation of complement activation	2.68E-41
Regulation of humoral immune response	2.58E-40
Complement activation	2.80E-40
Defense response	1.46E-36
Humoral immune response	1.49E-36
B	
Description	False Discovery Rate
Peptidase regulator activity	6.49E-25
Endopeptidase inhibitor activity	7.50E-25
Enzyme inhibitor activity	5.72E-21
Serine-type endopeptidase inhibitor activity	3.49E-16
Glycosaminoglycan binding	4.01E-16

Shared biological processes and molecular functions of serum proteins common between MStern and dHSP platforms analyzed by (A) Gene Ontology (GO) and (B) molecular function. Pathway analysis conducted through STRING DB.

Using the dHSP platform, we first identified those significantly different in abundance in LD sera at the time of infection in comparison to healthy controls (**Figure 1A**). Of the 46 differentially expressed proteins (DEPs) identified, many were involved in inflammation (complement and immunoglobulin molecules) and in innate immune regulation and tissue repair, including serum amyloid P, apolipoproteins, gelsolin and serpins. All but four of these proteins were also detected using the MStern platform (data not shown). Analysis of samples from subjects with localized infection identified 56 DEPs in comparison to healthy controls (**Figure 1B**). All other DEPs identified in the combined disease groups constituted a subset of those found in the localized infection group (**Figure 1A**). The additional 11 proteins found in the localized group were predominantly complement components and inflammatory markers (**Figure 1B**). In contrast, only 21 DEPs were identified in the disseminated LD samples as differentially expressed across all time points analyzed (**Figure 1C**), and were also observed as differentially expressed in localized infection. The lower number of DEPs in the disseminated LD samples in comparison to the localized LD samples may be due to the greater variability in subject presentations and evolution of responses over time in this subject group.

To better understand serum proteome changes in the localized and disseminated LD subjects, we compared proteomes longitudinally across these groups. We identified a small number of proteins whose expression changed significantly (Localized – C9: p=0.0161, AMBP: p=0.0127; Disseminated – CD44: p=4.19E-06, CFD: p=0.023) over time (**Figure 2**). The expression of most of these proteins exhibited a downward trend, approaching levels found in healthy controls over time. The exception was CFD, the rate-limiting enzyme required for the formation of C3 convertase and critical for complement activation *via* the alternative pathway.

At the time of initial presentation, the dHSP platform analysis identified 8 DEPs that distinguished between the localized and disseminated LD groups with significance assessed by p value. These proteins were all higher in the localized samples (C8G, KLKB1, AHSG, GSN, TTR, AFM, SerpinA7, and SerpinF2) and functionally are known to modulate the inflammatory response and innate immune pathways.

We next individually examined the data from the MStern platform characteristic of localized vs disseminated LD (**Figure S1A**) and reported DEPs and differentially expressed immunoglobulin domains (DEIgs). We identified C9 protein as decreasing in abundance in the localized group from the M0 (initial diagnosis) to the M3 (4-6 months after diagnosis) timepoints (C9; p = 0.011). In addition, MStern identified two other proteins demonstrating significant differences, C-reactive protein (CRP: p = 0.0026), and Inter-Alpha-Trypsin Inhibitor Heavy chain 2 (ITIH2: p = 0.03). A different isoform ITIH3 was identified on the dHSP platform. These proteins have been identified as being differentially regulated in LD in a previous study (7). While the proteins upregulated in localized LD were identified as mostly involved in the complement system, the proteins upregulated in disseminated LD at the initial timepoint (month zero (M0)) were involved in TLR regulation and hemostasis. Additional analyses at subsequent time points revealed a decline in numbers of DEPs in both.

We next used the STEM (short time-series expression miner) tool to visualize protein clusters and trajectories longitudinally in each LD subject group over the 3 time points independently (20) (**Figures S1B-D**). Three statistically significant clusters were identified within the protein trajectories of the disseminated LD samples, which were subsequently imported into the STRING interaction network to investigate their functional grouping. We then selected the same proteins and plotted their trajectories measured for the localized LD samples (**Figures S1B-D**). Very different trajectories of the same sets of proteins were apparent even though in both groups they resolved to be similar by the last time point. In the first cluster (**Figure S1B**), the protein dynamics for the disseminated LD group decreased from M0 to M1 to remain stable between M1 and M3. Some of these proteins are involved in the acute phase response (highlighted in red) and the complement cascade (highlighted in blue) from the STRING interaction network analysis. In the second cluster (**Figure S1C**), the protein trajectories demonstrate a continuous decrease across the three time points for the disseminated LD. Some of these proteins are involved in the negative regulation of proteolysis. Lastly, the third cluster (**Figure S1D**) showed a pronounced increase in protein intensity from M0 to M1 and a stability at M3. Some of these proteins are involved in the regulation of peptidase activity. It is noteworthy that the median trajectories for the same protein set show no change for the localized LD samples. These findings confirm the different proteome profiles observed between localized and disseminated LD from the acute infection to the resolution of signs and symptoms.

### Longitudinal proteomic profiles of responses in WNV

We profiled circulating proteins in the serum of our WNV subject cohort in samples collected longitudinally from acute infection (month 0) through convalescent time points at month 3 and month 12 after study entry. We identified 276 serum proteins with significant overlap (n=90) between the two proteomic platforms (**Figure 3C**). The MStern platform identified and quantified a total of 265 proteins of which 175 were unique to MStern, and an additional 11 unique proteins were identified by the dHSP platform alone. The Gene Ontology and molecular function pathways for proteins that are common between the two platforms feature elements relevant for complement activation, and humoral immune response regulation as well as regulation of peptidases (**Tables 3A, B**).

**Table 3:** Proteomic pathways in WNV.

A	
GO Description	False Discovery Rate
Regulation of complement activation	8.16E-41
Regulation of humoral immune response	2.94E-40
Complement activation	1.33E-31
Complement activation, classical pathway	2.46E-30
Regulation of immune effector process	1.92E-27
B	
Description	False Discovery Rate
Peptidase regulator activity	4.85E-24
Endopeptidase inhibitor activity	2.13E-23
Enzyme inhibitor activity	2.85E-22
Serine-type endopeptidase inhibitor activity	4.02E-19
Enzyme regulator activity	1.57E-18

Shared biological processes and molecular functions of serum proteins common between MStern and dHSP platforms analyzed by (A) Gene Ontology (GO) and (B) molecular function. Pathway analysis conducted through STRING DB.

**Figure 4:**
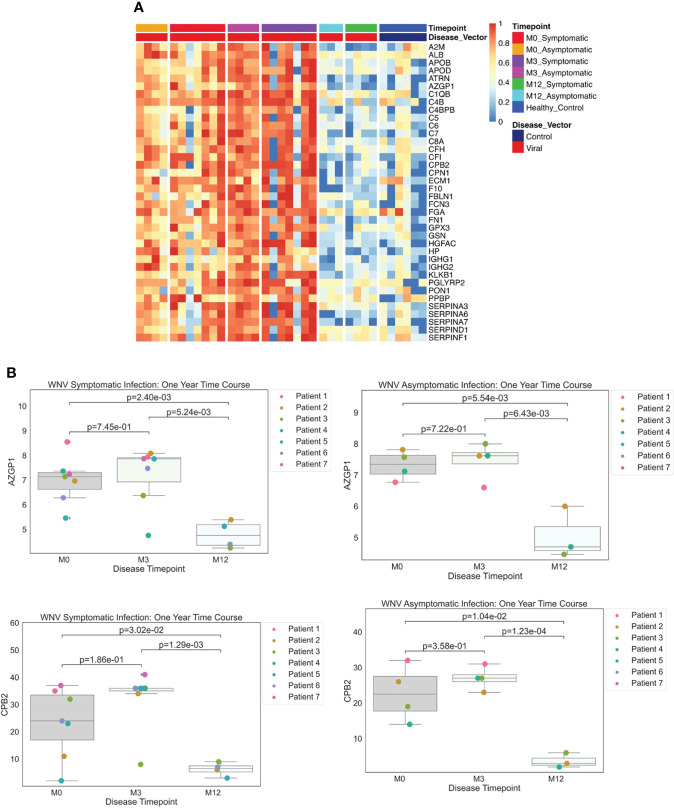
**(A)** Protein Expression across WNV Timepoints. Serum samples from WNV patients were assessed by the dHSP platform. The limma package was used to identify differentially expressed proteins in a combined dataset of healthy controls and patients with statistically significant proteins defined with FDR ¾= 0.05. A Normalized heatmap displays the progression of abundant proteins (n=38) in both symptomatic and asymptomatic infections at month 0, 1, and 3 (M0, M1, M3) compared to healthy controls. **(B)** Strip plots of differentially expressed proteins between primary acute infection and recovery in West Nile Virus. Serum samples from WNV patients were assessed by the dHSP platform. Differentially expressed proteins were identified in a combined dataset of acute infection and convalescent time points. Patients with statistically significant proteins were defined with p value ¾= 0.05. Both asymptomatic and infections were statistically significant across time points for C4B and CPB2.

Examining data from the dHSP platform, we compared serum proteins of WNV subjects that were differentially abundant at acute infection (month 0) in comparison to healthy controls and detected elevated levels of 38 significant WNV proteins (**Figure 4A** p < 0.05, FDR < 0.05). These proteins were associated with acute phase responses and inflammatory processes including the classical complement pathway proteins (C1QB, C4B, C4BPB, C5, C6, C7, C8A, KLKB1) and anti-inflammatory protease regulation pathways (Serpins A3, A6, A7, D1, F1) (27). When comparing proteomes across the time course, the convalescent samples collected at the 3-month time point showed sustained elevated levels of key proteins in both asymptomatic and symptomatic WNV subjects, indicating ongoing host proteome perturbations (**Figure 4A**). These significant changes in abundance of serum proteins and markers of inflammation highlight prolonged immune perturbations for at least 3 months after WNV infection. Notably, protein levels at the 12-month convalescent time point were not significantly different from healthy controls suggesting a return to baseline levels of circulating proteins (**Figure 4A**). Both asymptomatic and symptomatic cases of WNV showed a decrease over time in protein expression of alpha-2-glycoprotein 1 (AZGP1) (p-values of 0.0055, 0.0024 respectively), which plays a role in peptide antigen binding and carboxypeptidase B2 (CPB2) (p-values of 0.01 and 0.003 respectively), which cleaves C terminal residues from biologically active anaphylatoxins in the circulation (**Figure 4B**).

To identify proteins which may contribute to the divergence in the clinical course of WNV infection, we compared differentially abundant proteins between symptomatic and asymptomatic study participants. At the acute infection time point (month 0), we detected a significant elevation for a single protein in symptomatic compared to asymptomatic subjects: insulin like growth factor binding protein acid-labile subunit (IGFALS) was 1.8-fold higher (p<0.04, FDR <0.05) in symptomatic subjects.

As in our analysis of LD proteomes, serum samples from WNV subjects were assessed using the MStern blotting-based platform without depletion of the most abundant serum proteins. In comparison to healthy controls, all WNV subjects showed elevations in proteins involved in the acute phase response and inflammatory processes, including Siglec 16 (28), β-2 microglobulin (B2M), a component of class I major histocompatibility complexes which is elevated in viral infections (29), and the fibrinogen, complement, and coagulation pathways (27). Another highly elevated protein was apolipoprotein A1 (APOA1) which responds to inflammatory cytokines and is involved in autophagy (30).

The MStern blotting-based pipeline identified 30 proteins showing significant differential abundance over the time course and severity of WNV infection. In particular, we detected differential abundance of multiple apolipoproteins (APOA1, APOA2, APOA4, APOD, APOE, APOL1, APOM). The most significant DEPs were elevated at the acute time point in the most symptomatic patients. These include the vitamin D-being protein GC, plasminogen (PLG), an acute phase reactant; and APOL1. CD14, the soluble form of the monocyte surface marker, was also elevated in severe patients, and is an indicator of monocyte activation consistent with other elements of immune activation (31). These differentially abundant proteins are highly similar to those identified by the dHSP platform. In particular, apolipoproteins and SERPINS were identified as differentially abundant in both platforms. As noted above for the dHSP pipeline, the MStern blotting-based platform also detected IGFALS at elevated levels in symptomatic subjects. The consistent detection of this severity-distinguishing protein by both platforms underscores the robust differences in levels and suggests an important role in pathogenesis.

### Comparing LD and WNV proteomes

Our dual analyses allowed us to assess the similarities and differences in proteomic profiles between LD and WNV infections. When compared to healthy controls, on analyzing the dHSP data, the proteins that were identified to be differentially abundant across all time points in the LD and WNV sera overlapped considerably (**Figures 1A**, **4A**). Of the 46 proteins identified in LD (**Figure 1A**), 35 were shared with WNV samples. Eleven proteins were unique to LD (APOC1, C1r, C4bpa, C9, F13B, HRG, ITIH1, ITIH3, KNGisoform lmw, LRG1, and PROS1) and 3 were unique to WNV (ALB, ECM1, and FN1). When samples at the time of diagnosis were compared, 4 proteins (APOM, C4BPB, CPN2 and F11) were found to be in higher abundance in the LD samples compared to WNV.

We next used a random forest algorithm to determine which proteins contributed the most to the plasma proteome patterns of LD and WNV infections by the dHSP platform and also whether different subsets of the proteins shared in both diseases contributed most to distinguishing each infection from healthy controls. The random forest machine learning model used 70% of the data from LD and WNV for learning, preserving 30% for testing. We confirmed the differences between healthy controls and the LD and WNV subjects when compared at time points approximating similar periods of infection (M3 in LD and WNV). **Table 4** lists the proteins identified with random forest and determined by gini impurity as the most important in distinguishing all LD subjects from controls and **Table 5** lists those distinguishing all WNV subjects from controls. The random forest area under the curve (AUC) was determined to be 1.00 for LD and 0.94 for WNV.

**Table 4:** Proteins distinguishing LD from healthy controls.

Protein	Protein Name	Importance
ITIH3	Inter-Alpha-Trypsin Inhibitor Heavy Chain 3	1.051E-01
KLKB1	Kallikrein B1	5.798E-02
C1R	Complement C1r	5.780E-02
C4BPB	Complement Component 4 Binding Protein Beta	5.251E-02
C8A	Complement C8 Alpha Chain	5.118E-02
C7	Complement C7	5.020E-02
APOC1	Apolipoprotein C1	4.840E-02
HRG	Histidine Rich Glycoprotein	4.741E-02
PGLYRP2	Peptidoglycan Recognition Protein 2	4.576E-02
C6	Complement C6	4.514E-02
HP	Haptoglobin	4.159E-02
ATRN	Attractin	3.427E-02
C5	Complement C5	3.393E-02
SERPINF1	Serpin Family F Member 1	2.539E-02
F10	Coagulation Factor X	1.864E-02
FCN3	Ficolin 3	1.797E-02
IGFBP3	Insulin Like Growth Factor Binding Protein 3	1.576E-02
IGHG2	Immunoglobulin Heavy Constant Gamma 2	1.496E-02
SERPING1	Serpin Family G Member 1	1.289E-02
APOD	Apolipoprotein D	1.151E-02

List of important features that were pivotal in determining the difference between a Lyme Disease patient and healthy control when using a random forest model.

**Table 5:** Proteins distinguishing WNV from healthy controls.

Protein	Protein Name	Importance
C4A	Complement C4A	6.349E-02
C4B	Complement C4B	5.340E-02
FGA	Fibrinogen Alpha Chain	3.949E-02
GPX3	Glutathione Peroxidase 3	3.824E-02
PPBP	Pro-Platelet Basic Protein	3.638E-02
PGLYRP2	Peptidoglycan Recognition Protein 2	3.377E-02
AZGP1	Alpha-2-Glycoprotein 1, Zinc-Binding	3.364E-02
ITIH3	Inter-Alpha-Trypsin Inhibitor Heavy Chain 3	2.657E-02
SERPINA7	Serpin Family A Member 7	2.485E-02
HGFAC	HGF Activator	2.222E-02
APOB	Apolipoprotein B	2.211E-02
HP	Haptoglobin	2.118E-02
C4BPB	Complement Component 4 Binding Protein Beta	2.048E-02
APOC3	Apolipoprotein C3	1.965E-02
C8G	Complement C8 Gamma Chain	1.963E-02
C7	Complement C7	1.911E-02
SERPIND1	Serpin Family D Member 1	1.884E-02
CPB2	Carboxypeptidase B2	1.828E-02
APOA2	Apolipoprotein A2	1.773E-02
APOD	Apolipoprotein D	1.306E-02

List of important features that were pivotal in determining the difference between a WNV patient and healthy control when using a random forest model.

Although the majority of proteins identified as abundantly increased were found to be identical between the two diseases (**Figures 1A**, **4A**), in fact machine learning identified several differences in their importance (**Tables 4**, **5**). ITIH3 contributed most significantly to the differences between healthy controls and LD subjects but was not a major contributor for distinguishing WNV subjects from controls. In contrast, C4A contributed most significantly to distinguishing WNV from controls but did not contribute to distinguishing LD subjects from healthy controls. Of the top 20 contributing proteins for each infection identified by machine learning, 6 were found to be common to both LD and WNV (APOD, C4BPB, C7, HP, ITIH3, PGLYRP2), but with varying degrees of importance in each disease (**Tables 4**, **5**). **Table 6** lists the proteins identified with random forest and determined by gini impurity that are most pivotal to distinguishing LD from WNV subjects. The AUC for distinguishing LD from WNV subjects is 0.73, highlighting that larger cohorts are needed to ascertain whether serum proteomics can be used to develop panels that reliably distinguish these two vector-borne diseases.

**Table 6:** Proteins distinguishing LD and WNV.

Protein	Protein Name	Importance
FN1	Fibronectin 1	4.245E-02
AZGP1	Alpha-2-Glycoprotein 1, Zinc-Binding	4.126E-02
ITIH3	Inter-Alpha-Trypsin Inhibitor Heavy Chain 3	4.111E-02
KNG1IsoformLMW	Kininogen 1 Lower Molecular Weight Isoform	3.735E-02
RBP4	Retinol Binding Protein 4	3.138E-02
C1R	Complement C1r	2.786E-02
GPLD1	Glycosylphosphatidylinositol Specific Phospholipase D1	2.642E-02
CPN1	Carboxypeptidase N Subunit 1	2.234E-02
VTN	Vitronectin	2.209E-02
SERPINA1	Serpin Family A Member 1	2.083E-02
APOE	Apolipoprotein E	2.020E-02
APOD	Apolipoprotein D	1.990E-02
C4B	Complement C4B	1.952E-02
IGHG1	Immunoglobulin Heavy Constant Gamma 1	1.940E-02
PLG	Plasminogen	1.773E-02
AMBP	Alpha-1-Microglobulin/Bikunin Precursor	1.688E-02
TTR	Transthyretin	1.660E-02
SERPINA6	Serpin Family A Member 6	1.630E-02
HP	Haptoglobin	1.621E-02
IGKC	Immunoglobulin Kappa Constant	1.614E-02

Important features that were pivotal in distinguishing between LD and WNV were determined by gini impurity analysis.

## Discussion

Analysis of serum proteomic changes using mass spectrometry techniques is increasingly being used to define clinical biomarkers and to gain insight into potential mechanisms of disease. Our studies were conducted using two LC/MS based detection platforms, one a depleting platform (dHSP) that uses the approach of depleting highly abundant serum proteins to augment the detection of lower abundant, biologically significant proteins that may otherwise not be detected in human serum (4). The second method, the MStern platform, utilizes a custom generated protein detection platform, emphasizing rapid, low-cost and low-throughput detection, that could be applied for clinical validation, after deeper interrogations have been conducted. The MStern complements standard LC/MS platforms, as without depletion of highly abundant proteins, those with immunologic roles such as immunoglobulins and acute phase proteins are retained allowing for a more complete view of the immune status (32). The two platforms utilized in this study provided additive evaluations of serum protein measures and trends, and the independent platforms using identical samples are validation for the overlapping proteins identified.

Our study is novel as it applies these techniques to examine the evolution of the human proteome response to infection with two arthropod-transmitted pathogens that differ both in their vectors (tick vs mosquito) as well as their taxonomic classification – one a spirochetal bacterium (LD) and the other a flavivirus (WNV). Both infections elicit type I IFN responses and give rise to a spectrum of disease states (33–35). Although these pathogens are quite distinct microbiologically, we found similarities in the serum proteomes of infected subjects, beyond the known type I IFN signatures.

In LD, many of the proteins identified were involved in inflammation (complement and immunoglobulin molecules) and in innate immune regulation and tissue repair, including serum amyloid P, apolipoproteins, gelsolin, and serpins. Proteins identified distinguishing early LD by previous studies (7) were proteins also identified in our analyses (C9, ITIH2, PGLYRP2) along with related apolipoproteins, which further validates the LD findings in this study. These concordant results support the value of proteomic analyses and the prominent involvement of innate immune pathways and acute phase proteins in orchestration of initial pathogen defense and its regulation.

Infection with WNV induced expression of serum proteins associated with acute phase responses and inflammatory processes including complement pathway proteins C4B, and kallikrein (KLKB1), both of which are involved in complement activation (36). Both platforms also detected elevated levels of anti-inflammatory protease regulation pathways SERPINA3, a serine protease inhibitor with anti-inflammatory effects inhibiting PMN granule protease cathepsin G (37). Other elevated proteins include immunoregulatory factors which may play a protective role in limiting an overexuberant inflammatory response potentially contributing to permeability of the blood brain barrier and encephalitis. These pathways include apolipoprotein B (APOB) which has a structural role in LDL as well as limiting inflammation in infection (38); carboxypeptidase (CPB2) which functions to inhibit fibrinolysis; and immune related proteins such as zinc binding alpha-2 glycoprotein-1 (AZGP1), a glucocorticoid regulated fatty acid-binding protein with homology to MHC I molecules and a role in inhibiting the TGFβ signaling pathway (39). A single protein, the classical complement pathway factor C2, was lower in WNV infected individuals at acute infection (month 0) compared to healthy controls. While no global proteomic studies of human study participants with WNV infection have been reported to date for comparison, murine models suggest a critical role for complement in response to WNV infection (40). A lower level of C2 may be expected reflecting its consumption in response to the WNV infection (40).

A striking finding is that significant changes in the proteomes were present for several months after the initial infection despite clinical resolution of disease. For WNV, analysis of samples collected at 12 months after diagnosis revealed that most protein levels had returned to baseline levels present in healthy controls, suggesting that it takes longer than 3 months for proteomic evidence of infection in sera to resolve. Twelve-month convalescent samples were not available for the LD cohort, although our STEM analysis suggested that there is some convergence of localized and disseminated expression at 3 months of infection. An earlier transcriptional analysis of PBMCs of LD subjects documented sustained differential gene expression at 6 months after infection (18), consistent with ongoing immune perturbations.

Our study presents a novel profiling of proteomic responses to infection, however, substantial conclusions about the infectious course are limited by the small sample size in the exploratory study design of this report. Nevertheless, our bioinformatic and statistical data analyses allowed us to define features of the proteome and pathophysiological processes that can distinguish each infection from healthy controls. Using machine learning with random forest analyses, we found that the DEPs identified for each disease reliably distinguish infected individuals from healthy controls regardless of disease severity, with AUC’s of 1.0 and 0.94 for LD and WNV respectively. It also showed limitations, however, in using the proteome alone to distinguish between infectious diseases. The random forest analyses aimed at identifying distinct subsets of proteins that might distinguish LD and WNV infection could not be identified in this rather small number of patients in each infection group. These results together with the identification of largely overlapping lists of DEPs from each disease further support the conclusion that acute responses to microbiologically distinct infections transmitted by different vectors induce very similar serum proteome responses. Moreover, these responses persist for at least three months post infection, independent of initial disease severity, even when clinical data indicate that the disease signs have largely resolved.

Some of the proteins that were elevated for at least 3 months post-infection in LD subjects or in both LD and WNV participants have also been differentially abundant in other human infections. F13B (coagulation), HRG (platelet degranulation) and APOC1 (lipid metabolism) found in lower levels in patients with severe COVID-19 infection with lowest amounts in those whose disease has progressed (41). Interestingly, we find the opposite in LD subjects, who have higher abundance of these proteins than controls. GSN (coagulation) is also down regulated in COVID-19 patients with increasing severity correlated with the lowest levels. GSN is in higher abundance in both LD (in particular the localized disease subjects) and WNV infection for at least three months. There are also similarities between LD subjects and severe COVID-19 subjects with KNG (pro coagulation-platelet degranulation), LRG1 (antimicrobial), and PROS1 (anticoagulant), which are found in higher abundance in both. The protein IGFALS, which distinguishes severity of WNV infection, facilitates ubiquitination of signaling mediators IRAK1 and TRAF6 to inhibit influenza viral replication and has recently been shown to be a sensitive biomarker of COVID-19 infection (42, 43).

In summary, the substantial overlap in DEP between LD and WNV study participants regardless of their disease presentation suggests that serum proteomic profiles may hold important clues about acute inflammatory responses that are more generalizable than was previously thought. Advances in proteomics are rapidly revealing multiple unexpected functions of circulating proteins such as APOL3, recently demonstrated to have detergent-like antibacterial activity beyond its expected role in cholesterol transport (44). Future studies may identify common pathways and processes induced by a variety of pathogens—rather than disease-specific profiles–which may be targets for disease modifying interventions.

## Data availability statement

The datasets presented in this study can be found in online repositories. The names of the repository/repositories and accession number(s) can be found below: https://immport.niaid.nih.gov/home, SDY1396 - Experiment 33420, SDY 1397 – Experiment 33421, SDY1394 – Experiment 34003, SDY1395 – Experiment 34004.

## Ethics statement

The studies involving human participants were reviewed and approved by Human Investigations Committee of Yale University School of Medicine (LD) and Baylor College of Medicine (WNV). The patients/participants provided their written informed consent to participate in this study.

## Author contributions

Manuscript was drafted by PB, TS, MS, BF, KS, HS, RM, AB, OL; RM, LB designed the clinical study; AB and LB were responsible for LD sample collection, handling and distribution including clinical data capture; RM, SR, KM were responsible for WNV sample collection, handling and distribution including clinical data capture; BF, HS, PB, TS, MS designed the analytical strategy; BF, KS, ZW, HS were HS were responsible for the MStern blotting-based processing and analysis of the samples; JL, PR, MK, TS, PB, MS were responsible for the processing and analysis of the samples using the dHSP pipeline; BS, KS, HS, PB, TKS, MS analyzed the data. All authors listed above contributed to the data analysis and/or interpretation and reviewed and approved the manuscript including the final version as submitted.

## Supplementary material

Supplementary Figure 1 Proteome comparison in localized and disseminated LD participants over time. **(A)** Analysis of differentially expressed proteins (DEPs) and immunoglobulins (DEIgs) between the localized (orange) and the disseminated (blue) LD samples at M0 **(A)**, M1 **(B)** and M3 **(C)** time points. For each cluster, the protein names were exported into the STRING interaction network. The white dotted lines represent the median of the Log2 difference of all proteins in the cluster. The proteins highlighted in red are involved in the acute phase response, in blue the proteins involved in the complement cascade, in green the proteins involved in the negative regulation of proteolysis, in yellow the proteins involved in the protein activation cascade, and in pink the proteins involved in the blood coagulation. **(B-D)**. Longitudinal analysis using the STEM program for clustering of the averaged protein intensity profiles across the 3 time points for the disseminated LD group and applying the protein cluster to the localized LD group.
