# Supplementary figures and images for: Longitudinal serum proteomics analyses identify unique and overlapping host response pathways in Lyme disease and West Nile virus infection
Source: Front Immunol. 2022 Dec 9;13:1012824. doi: 10.3389/fimmu.2022.1012824 (PMC9784464; doi:10.3389/fimmu.2022.1012824)

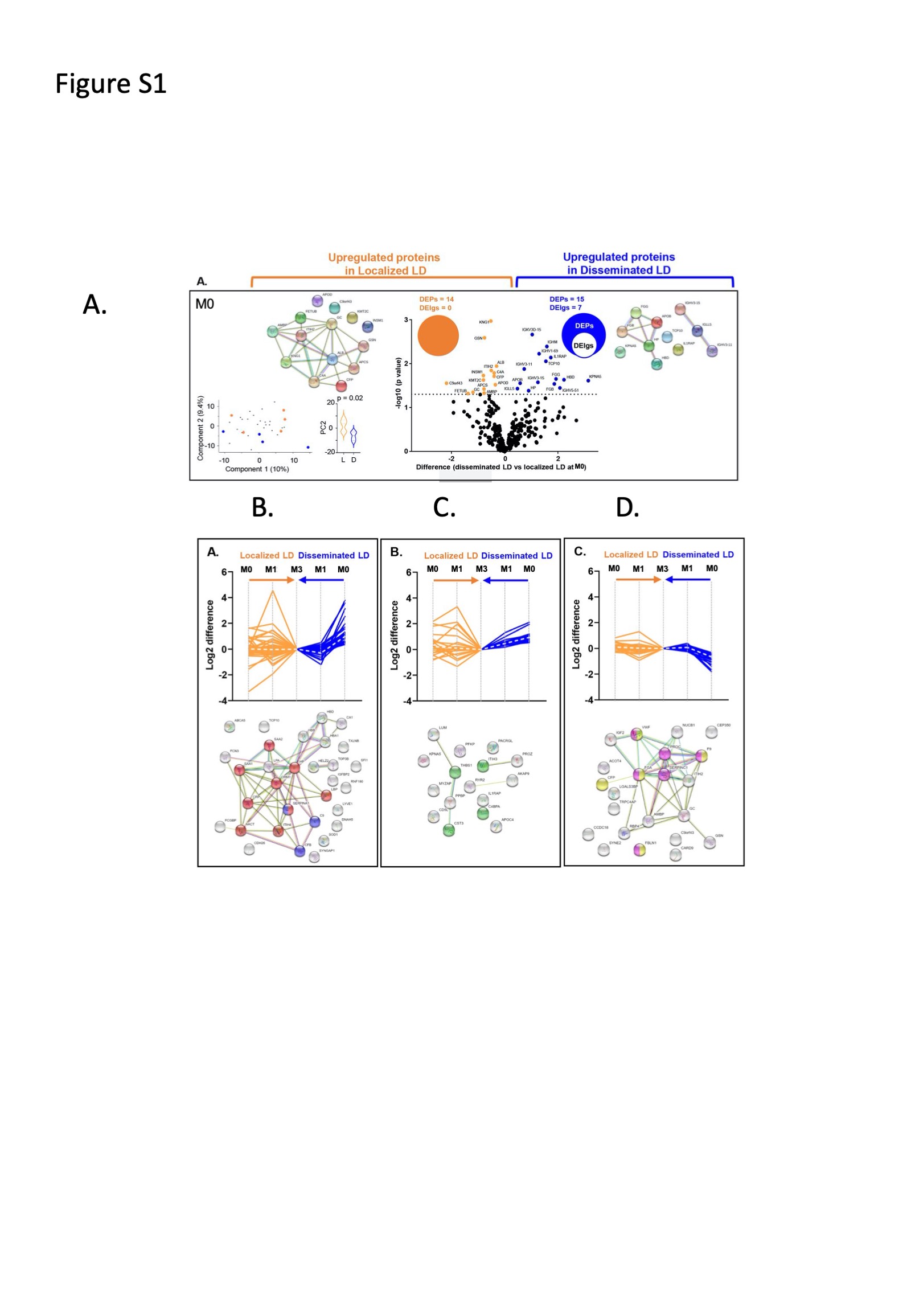

Supplement: Supplementary Figure 1 — Proteome comparison in localized and disseminated LD participants over time. (A) Analysis of differentially expressed proteins (DEPs) and immunoglobulins (DEIgs) between the localized (orange) and the disseminated (blue) LD samples at M0 (A), M1 (B) and M3 (C) time points. For each cluster, the protein names were exported into the STRING interaction network. The white dotted lines represent the median of the Log2 difference of all proteins in the cluster. The proteins highlighted in red are involved in the acute phase response, in blue the proteins involved in the complement cascade, in green the proteins involved in the negative regulation of proteolysis, in yellow the proteins involved in the protein activation cascade, and in pink the proteins involved in the blood coagulation. (B-D). Longitudinal analysis using the STEM program for clustering of the averaged protein intensity profiles across the 3 time points for the disseminated LD group and applying the protein cluster to the localized LD group. [file Image_1.jpeg]
